# From virtual to real healing: a critical overview of the therapeutic use of virtual reality to cope with mourning

**DOI:** 10.1007/s12144-021-02158-9

**Published:** 2021-08-20

**Authors:** Silvia Francesca Maria Pizzoli, Dario Monzani, Laura Vergani, Virginia Sanchini, Ketti Mazzocco

**Affiliations:** 1 Department of Oncology and Hemato-Oncology, University of Milan, Via Festa del Perdono 7, 20122 Milan, Italy; 2 Applied Research Division for Cognitive and Psychological Science, IEO, European Institute of Oncology IRCCS, Milan, Italy

**Keywords:** Virtual reality, Virtual therapy, Grief, Bereavement, Mourning

## Abstract

In recent years, virtual reality (VR) has been effectively employed in several settings, ranging from health care needs to leisure and gaming activities. A new application of virtual stimuli appeared in social media: in the documentary ‘I met you’ from the South-Korean Munhwa Broadcasting, a mother made the experience of interacting with the avatar of the seven-year-old daughter, who died four years before. We think that this new application of virtual stimuli should open a debate on its possible implications: it represents contents related to grief, a dramatic and yet natural experience, that can have deep psychological impacts on fragile subjects put in virtual environments. In the present work, possible side-effects, as well as hypothetical therapeutical application of VR for the treatment of mourning, are discussed.

I met you

“Mom, did you think about me? I miss you a lot”.

“Mom missed you too”.

These are the first words of the touching and virtual reunion of a South Korean mother and her seven-year-old daughter who recently passed away. With the help of virtual reality (VR) technology, a team of experts made it possible for this young mother to interact with a virtual avatar of her daughter, talking with her and even song her “Happy Birthday” before blowing off seven candles on the birthday cake. “I met you”, the documentary describing this moving experience produced by South-Korean television network MBC Life, has rapidly reached millions of views worldwide as well as suggesting a possible new application of virtual stimuli.

## Grief Process and Virtual Reality

In recent years, virtual reality (VR) has been effectively employed in a wide variety of settings, ranging from leisure and gaming activities to health care needs (Freeman et al., 2017; Tudor Car et al., 2019). The possibility of creating virtual stimuli is potentially limitless and it continues to expand.

The documentary is an example of a novel application of personalized virtual content, strictly related to emotions and life events (Pizzoli et al., 2019). By subjecting the user to a highly relevant virtual stimulus, the former consequently lives a strong emotional experience.

Emotional engagement is somehow the “magic ingredient” of the virtual experience. Indeed, beyond the different aims of its application, the power of VR is making users feel as they were really present within virtual environments (VEs) (Slater, 2018). VR can produce the illusion of VEs, stimulating senses and presenting stimuli that in some cases could not have been reproduced in real settings, ranging from anatomical representations for medical purposes to fairy creatures in video games. The chance to interact with ‘virtual creatures’ not only involves a sense of presence but can also have deep psychological impacts. An example is represented by virtual reality exposure therapy (VRET), where the scope of the intervention is to make patients meet intensely feared objects and develop coping strategies to manage panic and distress (Wechsler et al., 2019). The emotional engagement in these cases is strong and therapeutically provoked.

The recent implementation of VR saw in ‘I met you’ further emphasizes the possibility of provoking emotional reactions, with a new category of virtual stimuli, namely personal contents related to dramatic and yet natural experiences (i.e., the loss of a beloved one), that can have a deep psychological impact. In nature, an experience that resembles the one recreated in ‘I met you’ is the phenomenon of hallucinations after bereavement, where a person, who does not suffer from psychiatric or neurological disorders, experiences hallucinations related to the deceased ones, seeing, hearing, feeling, tasting, smelling and sensing their presence (Grimby, 1993; Olson et al., 1985; Rees, 1971). These experiences are usually perceived as comforting ones (Castelnovo et al., 2015), but they correlate with symptoms of distress, such as severe conditions of loneliness, crying and memory issues (Grimby, 1993). Re-experiencing features of a beloved deceased one seems to give solace to suffering. Feeling the presence of someone we lost seems indeed to give a break to the pain of permanent loss and nostalgia.

Bereavement is a difficult experience per se, which challenges individual resilience (Bonanno, 2009) and requires passing through complex phases. As described by Elisabeth Kübler-Ross (Kübler-Ross, 1969), bereavement often moves from initial denial through acceptance and recovery, passing through anger, bargaining, and depression. This process takes time and pain. Not surprisingly, in traditional vis-a-vis psychological therapy, the mourning of a beloved one is often reported by the patient as one of the major causes of pain and suffering. Indeed, grief experience takes a hard process to reach recovery and can differ in length and intensity, depending on individual differences (Stroebe et al., 2006).

Different people may make use of different strategies: some might be engaged in approach behaviors (e.g. searching for and spending time with objects related to the deceased one), while some others might find it more comfortable to avoid reminders of the expired (Parkes & Prigerson, 2013). Furthermore, substantial individual differences regarding the extent of pain and the time spent adjusting to loss exists. Most people experience grief in a period ranging from weeks to months (Bonanno et al., 2002), and acute grief naturally evolves into integrated grief, where the person regains the ability to re-engage with everyday life and find interest or pleasure (Shear et al., 2013).

However, for a subset of individuals grief could manifest itself causing functional impairment even for years after the bereavement (Kersting et al., 2011). Such a protracted advancement of grief symptoms has been defined in several ways, from traumatic (Horowitz et al., 1997), to prolonged (Prigerson et al., 2009), and complicated grief (Shear et al., 2011). Lately, the American Psychiatric Association named such a situation as a persistent complex bereavement disorder (PCBD) (APA, 2013), while the ICD-11 classification system defined the disturb of grieving as the prolonged grief disorder (PGD) (Killikelly & Maercker, 2017). In such a condition a patient might manifest maladaptive behaviors (extreme avoidance of reminders of the lost person or disproportionate closeness seeking to the death one) and emotion dysregulation (anger about the death, persistent sensation of shock), as well as dysfunctional cognitive activation (ruminations, disbelief of the death). In extreme cases, desire to die and suicidal thoughts and behaviors (Shear et al., 2011). The process of grief shares some similarities with trauma reactions (Solomon & Rando, 2012), and it can also prompt major depressive disorder, posttraumatic stress disorder, and substance use disorders (Galatzer-Levy & Bonanno, 2012; Reynolds et al., 1999; Zisook et al., 2010). Furthermore, bereavement can affect physical health too and it can increase the risk of mortality (Stroebe et al., 2007).

Different methods can be employed to try to manage the mourning: psychoeducation on emotions and the phases of loss (Boelen et al., 2007; MacCallum & Bryant, 2011); Eye Movement Desensitization Reprocessing (EMDR) (Cotter et al., 2017; Solomon & Rando, 2012); cognitive-behavioral techniques and so on (Bergman et al., 2017; Cotter et al., 2017; Lenferink et al., 2017; Linde et al., 2017; Rosner et al., 2018). Apart from the chosen therapeutic approach, because of individual differences, therapy should create the opportunity to focus on the specific modality of grief and to tailor the intervention to specific needs. For instance, EMDR might help in recovering from traumatic memories related to the dead one, while cognitive-behavioral techniques (imaginary or expressive ones) might help in managing avoidant or approach behaviors; with cognitive expressive and imaginary techniques, therapists can try to help patients engaged with avoidant behavior to safely interact with the dead person, to express feelings and thoughts. Indeed, to date, cognitive-behavioral techniques and psychoeducation are the most effective approach to treat complicated grief (Simon, 2013), and there is mounting evidence about the utility of asking the bereaved person to continuously repeat the story of the death (Boelen et al., 2007; Wagner, 2006).

Therapists can also invite the mourner to bring to therapy session objects related to the deceased (as photos, audiotapes, or video clips) because it helps to create a sense of closeness to the deceased, which in turn facilitate the patient in process of expressing himself/herself and talking to the deceased rather than speaking about him or her (Worden, 2018).

In all these cases, the interventions aim to help patients to interact with memories and emotions related to the deceased person, to cope with the pain, and accept the loss. Namely, to elaborate the bereavement at the best they can. It obviously takes time and pain and sometimes the best result that might be achieved is a partial elaboration of the loss. As Simon (Simon, 2013) wrote, “*Because loss is forever, so too is the state of being bereaved*”.

## Possibilities and Potential Side Effects of a VR for Grief

The case of the documentary ‘I met you’ should be discussed by the scientific community and by health care professionals, as it proposed a strong experience that can have a significant and possible detrimental psychological impact on mourners. Which are the possible impacts of this new application of VR? Can it be a new therapeutic application of VR in the case of grief experience? How VR can be employed to deal with that experience of permanent loss?

Indeed, the experience with this kind of virtual avatars can be extremely emotional since the individual can interact with the lost person and can perceive the situation to be real because of immersion and presence. Such an experience is different from common behavior in grieving such as looking at photos or trying to be close to objectives and places associated with the beloved one since there can be no real-time interactions with photos or places, that are static stimuli and not animated avatars.

Furthermore, the excessive search for closeness to the death one and related objects might be a sign of a difficult pattern of grieving. Making an intervention that renders avatars of the deceased available might catch the attention of people who suffers from complex grieving and that might search for these interventions to fulfill a dysfunctional need.

In the following part of the paragraph, we aimed at describing possible key points to be addressed to plan an intervention with VR for grief as a therapeutic means.

Firstly, in our opinion, to be defined as “therapy”, a VR intervention should have a clear aim, built taking into consideration possible harms for the users. It should also be provided not as an open-source tool, but rather it should be available as a means to be used by and under the supervision of experts in the field of mental health, which can look after possible side effects on the psychological well-being of the users.

It seems that the objective of the documentary would be to lead a mother to meet the deceased daughter to interrupt the agony of the grief. This can be an experience much stronger than what can be provided in classical settings. Consequently, it can lead to enhanced effects and risks. In particular, the experience seems to lead to a peaceful closure experience for the mother who, after listening to a letter written by her daughter, can tell her what seems to be sort of goodbye words. However, even if the mother described the experience positively immediately after, we do not know the possible long-term consequences of that. It might be possible a person would become addicted to the relief given by the meeting with a lost one. We also do not have information about the mother’s phase of grief, strategies to cope with the pain, and general psychological state.

In the case of VRET, where patients are exposed to forcefully feared objects, therapists control exposure intensity to reach a clinical mean and to protect patients from over-exposure and stress (Boeldt et al., 2019). Furthermore, patients and therapists previously assessed together which is the minimum and the maximum degree of exposure they can tolerate, with the aim of not causing any psychological harm with the exposure. The same reasoning should be applied to the experience of re-met a deceased person, where an objective can be for instance the possibility of saying something to the lost one and of going on with the elaboration of the loss, as in the imaginary exposure techniques. In the cited documentary, a powerful virtual stimulus was used, an avatar of a lost child. However, future interventions might consider using less emotional stimuli, such as reproduction on objects or places linked to the deceased, used specific aims, as trying to expose the person to non-elaborated situations. The case of VRET can be used as a source of insight for future intervention for grief also because of the attention that it is paid to the choice and personalization of the stimuli to be put inside the VR. A careful process of personalization, based on the objective of the intervention, the tolerance of pain expressed by the users, and on users’ personal accounts of relevant autobiographical stimuli might strengthen the efficacy of VR for grief and reduce the risk of causing harm to participants. The importance of personalization has been increasingly stressed in the field of VR interventions, in particular in relaxation (Heyse et al., 2019; Heyse et al., 2020; Pizzoli et al., 2019) and PTSD treatment (Baños et al., 2011; Botella et al., 2010). Interestingly, possible target persons for VR for grief might be those who present symptoms that resemble post-traumatic or anxious symptoms, which tend to be chronic in the 40% of the bereaved subjects (Zisook et al., 1998) and that is more likely to be present after violent deaths (Kaltman & Bonanno, 2003).

Considering pre-existing approaches, to further assure the safeness of the future users of VR for grief, psychophysiological monitoring systems might be included in such interventions. This inclusion has been already done in the case of adaptative physiological systems in protocols on phobias, stress, and PTSD (Ćosić et al., 2010; Gaggioli et al., 2014; Petrescu et al., 2020), or biofeedback in relaxation (Blum et al., 2019, 2020).

Another important thing to consider is the Uncanny Valley effect (Moore, 2012), a term coined by Masahiro Mori in 1970, that indicates that VR stimuli used could cause a sense of revulsion and negative feelings in the user, especially if the artifacts are moving, like in the case we considered. We think that VR stimuli should be designed considering this possible effect, as well as clinicians, should be aware of it, to act and counteract its negative consequences for patients.

To assess the suitability of users and to avoid possible psychological side effects, those interventions should be carried out and programmed by an expert in the field of psychiatry or psychology. Indeed, professionals can offer a careful assessment of the possible risks and benefits linked to the interventions, as well as an evaluation of the specific grieving phase and the co-occurrence of grief with other disorders.

Bereavement is a severe life stressor, and it can trigger the onset of a physical or mental disorder. To protect possible users from hurt, personality and coping strategies should be thoughtfully screened, as well as the possible presence of a pathological condition related to grief. Indeed, it might occur in the case of a person with bereavement disorders (PGD or PCBD) asking for such an intervention to try to alleviate the pain, as we know that in some cases the desire for proximity to the deceased could be a symptom of this condition (Field, 2006). Among the grieving people, the prevalence of the PGD, as described by the ICD-11 (Killikelly & Maercker, 2017), is 9.8% (CI 6.8–14.0) (Lundorff et al., 2017). In this regard, the use of valid and reliable tools for the assessment of the disorders related to grief is fundamental not to include fragile participants. To date, a recent review identified 11 assessment tools aimed at identifying PGD or PCBD (Treml et al., 2020). Notably, there are no assessment instruments for the complete diagnostic assessment of the PGD, while there are three instruments for the PCBD (DSM-5 criteria) (the Persistent Complex Bereavement Inventory (Lee, 2015), the SCI-CG for DSM-5 (Bui et al., 2015), and the Traumatic Grief Inventory Self-Report Version (Boelen & Smid, 2017)). For the implementation of VR interventions for grief, we recommend the use of one of these three, paired with the informed evaluation by a clinician, to exclude the possibility of enrolling fragile participants in the experimental trial of this new approach.

Overall, before making such an intervention, the type of grief, the presence of PGD or PCBD, as well as personal resources and vulnerabilities of the target person should be carefully assessed. Currently, we do not know which consequences of VR for grief might occur for a person who is struggling with maladaptive behaviors, emotions, and thoughts related to grief.

Finally, preliminary controlled studies should be carried out to try to tailor interventions for recovery from grief, to test the suitability of users, specific activities, and scenarios.

As an initial step, future studies might test the feasibility and utility of insert simple stimuli related to the bereavement (such as objects or places) to try to target anxious and post-traumatic symptoms with a process already validated like extinction (Myers & Davis, 2007; Raeder et al., 2020). Furthermore, using techniques that are already employed in traditional settings might reduce the likelihood of harm for the patients or, at least, it should cause a tolerable dose of stress, as it is done in the case of exposure therapies and extinction for specific phobias (Choy et al., 2007; Eaton et al., 2018; Raeder et al., 2020), where patients have to confront with feared and distressing stimuli, with the long-term scope of alleviating anxiety. Importantly, each one of these steps should be carried out by qualified scientific institutions (universities and/or hospitals) and carefully revised and approved by Institutional Review Board.

As the experience with an avatar that resembles a lost person can be strongly emotive, it is difficult to plan future studies aimed at testing the feasibility and effectiveness of such stimuli. An a priori estimation of the possible harms and benefits would be very difficult since there is no available evidence on the effects of avatars in the treatment of grief.

In 2019, the construction of VR as a meaningful therapeutical tool was addressed by a panel of experts (Birckhead et al., 2019), which concluded that VR clinical studies should be divided into three phases. The first with studies aimed at content development by a human-centered design applied on patients and providers, the second one aimed at assessing the overall feasibility and the promises on clinical effectiveness, and the last phase composed by full powered randomized controlled trials for the evaluation of efficacy. Such recommendation practices should be carefully followed even in the case of VR for grieving.

For now, the VR application shown in the documentary resembles more interventions such as relaxation experiences or leisure games, where VR’s scope is to provide an immediate and short-term pleasing experience. It seems not to target resilience or recovery while creating the risk of causing extreme additional distress.

### Ethical Issues

In addition to psychological considerations, the scenario just described, and its different applications seem to raise ethical criticalities, therefore suggesting that ethical considerations should be made. First, we may wonder whether the emotional experience involved in VR when applied to this new context may be most beneficial or not. In a quite recent piece, Holly Brockwell seems to suggest that, in some specific cases, despite not being able to keep the beloved one alive, VR may still prove to be useful in allowing the individual to put an end to a still open situation, perhaps allowing a young man to say some leave words to a dad too early and suddenly disappeared (Brockwell, 2020). Even in this very specific case, doubts still exist as we may question whether this request may be considered as a step further in the grief process, or whether it is only another way to somehow trying to escape human finiteness, where it is not always possible to act according to our – even deepest – wishes. Additional complexities may raise if we consider that VR is used for a category of agents who are intrinsically vulnerable and, therefore, VR may create additional vulnerabilities than the ones they already possess (Sanchini & Marelli, 2020; Vines et al., 2017). These – only briefly mentioned – ethical considerations nevertheless suggest that VR seems to be qualitatively different from similar technological tools, in particular in terms of experiences the former generates (Ramirez & LaBarge, 2018). Therefore, case-by-case careful consideration and intense ethical scrutiny are required.

### Public Opinions

In a very short amount of time, the documentary has gained incredible popularity: while its views continue to grow, a countless number of articles appeared in newspapers and an increasing number of lay people comments on the video worldwide, on websites, forums, blogs, and social media. The reason for this popularity could be simple: losing a beloved one is a common, natural, and inevitable experience that concerns all of us and that is now facing the encounter with new technologies. However, while most people could relate to the South Korean mother’s grief and desire to meet her beloved deceased again, not all people are willing to live such an experience. From an initial and “bird’s eye view” look at laypeople comments on the web, these opinions seem to be various, heterogeneous, and wide-ranging: some pleased with this use of new technology, wishing to live a similar experience with a beloved dead one, others more critically point out the eventually negative consequences, like dependence or the stop of the natural process of death elaboration.

Using social media big data in a health context is increasing, also to understand public opinions (Conway & O’Connor, 2016) and, in this particular case, could be important: such opinions should be carefully taken into account, in addition to broader systematic studies, to tailor possible grief interventions according to peoples’ preferences and psychological features.

### COVID-19 and Funerals

Italy has been one of the first countries to be affected by the COVI-19 pandemic, with an increase in the number of deaths of 38,7% (from 65.592 to 90.946) in the period between the end of February and the end of March, compared to the mean of the same period of the past 4 years (INSTAT, 2020). One of the emblem images of the Italian COVID-19 Pandemic is a long line of military trucks that carried hundreds of coffins from the city of Bergamo, one of the most affected by the virus, to other crematoriums and cemeteries. During the first phases of lockdown, March and April 2020, people lived a peculiar psychosocial situation related to sanitary restrictions that impacted the experience of grief too.

For all the deaths which occurred in this period, regardless of the cause of the demise, it has been prohibited to do what is normal to do in case of loss: families and friends could not access the hospital to visit their beloved ones before or after the death; public funerals were forbidden, and the caskets were taken to cemeteries or crematorium; local cemeteries were closed to the public.

In these conditions, the experience of grief significantly changed, while funerals and rituals – that are related to the ability to cope with the loss (Mitima-Verloop et al., 2019) – were not possible. In the case of hospitalized patients for severe and long-term conditions such as cancer diseases (Marzorati et al., 2016), coping with trauma and changes might become further difficult. Consequences of this situation could be important, in the acute phase and in the long-term period. For example, among the risk factors for poor bereavement adjustment, there are sudden or traumatic death, a death in hospital, and the lack of social support (Lobb et al., 2010; Lundorff et al., 2017; M. Stroebe et al., 2007; Wright et al., 2010), all factors present in the COVID-19 Pandemic situation. Some authors already pointed out the necessity to manage bereavement during social isolation with effective strategies (Mayland et al., 2020; Morris, Moment, & Thomas, 2020; Wallace et al., 2020).

In this context, the possibility of reproducing some early and unlived phases of the griefs with VR might help bereaved people to have experiences they could not live because of the social isolation (such as funerals or last words to the beloved one). The application of VR, in this case, would have a specific curative aim and would target an unmet need that it has been impossible to satisfy in the real world in such situations. In this case, the steps and cautions described in the previous sections might provide some guidelines to prevent the psychological harm of such a new approach.

## Conclusions and Future Directions

We believe that studying the specific benefits and guidelines for VR interventions on grief could enhance the potential benefits of creating virtual experiences, useful in different contexts, with thoughtful attention to the deep psychological impact that such an experience might have and with a comparison and integration with the already existing approaches to treat bereavement. To date, the major issues related to the application of VR for grief are 1) the lack of clinical hypotheses and protocols, 2) the need for a careful assessment of the possible psychological risks related to such experience, 3) the lack of information about possible harms in the case of users with mental health struggles; 4) the need of understanding public opinions, tailoring these interventions to individual characteristics. Considering the lack of evidence on possible harms of VR for grief, we believe that, at the current state, grieving persons might be treated with VR interventions that target specific traumatic processes, as it has been effectively done for anxious disorders (Powers & Emmelkamp, 2008), and only inside a traditional therapeutic pathway. Indispensable elements should be the involvement of a health professional, with expertise in the field of trauma and grieving, and which should personalize the interventions according to the specific psychological needs of the user and which can assess specific harms and benefits linked to the exposure of virtual contents related to grief.
